# Illuminating what lies in darkness: Circadian regulation of hypocotyl growth in Arabidopsis via ELF3 recruitment of demethylases

**DOI:** 10.1093/plcell/koaf018

**Published:** 2025-01-16

**Authors:** Julie Robinson

**Affiliations:** Assistant Features Editor, The Plant Cell, American Society of Plant Biologists; HudsonAlpha Institute for Biotechnology, Huntsville, AL 35806, USA

Do you ever get so sleepy at night that you literally can’t keep your eyes open, even though you really want to finish watching the show you’re hooked on? Those heavy eyelids are the result of your body's circadian clock, which keeps you on a 24-hour cycle of wakefulness and sleep. Plants also rely on their circadian clock to coordinate biological functions, including those that promote the light reactions of photosynthesis only during the day when sunlight is available. The hypocotyl grows primarily during the night, and this, too, is regulated by the circadian clock in Arabidopsis (*Arabidopsis thaliana*; Niwa et al. 2009). Circadian clocks are composed of many different proteins, many of which operate in a complex system of overlapping negative feedback loops (Sanchez and Kay 2016).

Key players in plants’ circadian clocks are phytochrome-interacting factors (PIFs), transcription factors that transduce external light signals received by phytochromes, among other signals, into specific control of gene expression (de Lucas and Prat 2014). PIF4 and PIF5 repress hypocotyl growth during the day, while the evening complex enables hypocotyl growth during the night by repressing *PIF4/5* transcription. In new work, **Shiyu Tian and colleagues (Tian et al. 2025)** seek to elucidate the molecular mechanism by which the evening complex regulates *PIF4/5* expression.

Two key components of the evening complex are EARLY FLOWERING 3 (ELF3) and its interaction partner ELF4. Tian et al. performed a yeast three-hybrid screen to identify binding partners of ELF3 in the presence of ELF4. Through this screen, they identified JMJ17 and JMJ18, which are known members of a family of demethylases known as Jumonji (JMJ) proteins. The authors go on to characterize the role of JMJ17/18 in the light-dependent regulation of hypocotyl growth in Arabidopsis by investigating which gene region(s) of *PIF4*/*5* the demethylases bind to and the role of ELF3 in that interaction. They find enrichment of H3K4me3 within *PIF4*/*5* in both *elf3* and *jmj17jmj18* mutants, suggesting that the association of the demethylases with *PIF4/5* depends on ELF3.

Chromatin structure around a gene affects how well transcription machinery can access the gene to transcribe it. Methylated H3K4 is generally associated with actively transcribed genes (Hyun et al. 2017), and indeed *PIF4*/*5* genes are actively transcribed when methylated. Thus, the interaction of the demethylases JMJ17/18 with these genes has a significant effect on their expression, as summarized in the model presented by the authors (Figure). During the day, the evening complex is not formed and thus does not recruit the demethylases JMJ17/18 to *PIF4/5*, thus leaving them methylated. In their methylated state, they are actively transcribed, and the downstream effect of this is the prevention of hypocotyl growth. During the night, however, the evening complex recruits JMJ17/18 to *PIF4*/*5* through the action of ELF3, resulting in the demethylation of *PIF4*/*5*. When demethylated, transcription of these genes is repressed, which has the downstream effect of allowing hypocotyl growth. Interestingly, the authors also found significantly low expression of *PIF4/5* during the day in *elf3* despite significantly enriched H3K4me3 at *PIF4/5* at this time; epigenetic effects may explain this discrepancy.

**Figure.**
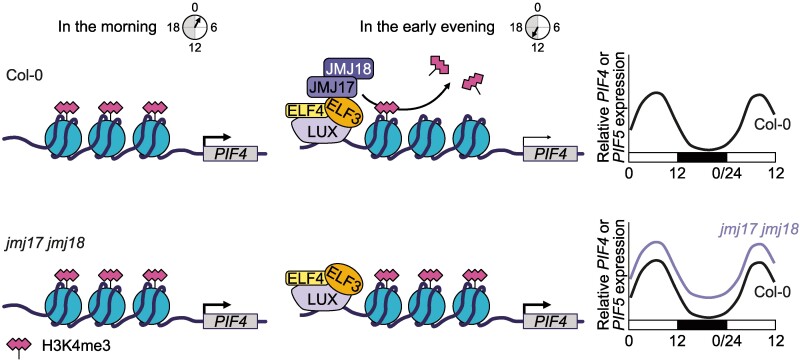
During the night in wild-type Arabidopsis (Col-0), the evening complex (EC) recruits JMJ17 and JMJ18 to demethylate H3K4me3 at *PIF4* and *PIF5*. This demethylation represses transcription of *PIF4* and *PIF5*, thus allowing hypocotyl growth. Reprinted from Tian et al. (2025), Figure 8.

The next evening you feel sleep overtaking you, know that seedlings the world over are just beginning to put their energy toward hypocotyl growth thanks to two JMJ demethylases under the direction of the evening complex.

## Dive Curated Terms

The following phenotypic, genotypic, and functional terms are of significance to the work described in this paper:

PIF4 Gramene: AT2G43010
PIF4 Araport: AT2G43010

ELF3 Gramene: AT2G25930
ELF3 Araport: AT2G25930

ELF4 Gramene: AT2G40080
ELF4 Araport: AT2G40080

PIF5 Gramene: AT3G59060
PIF5 Araport: AT3G59060
